# Sex impacts cardiac function and the proteome response to thyroid hormone in aged mice

**DOI:** 10.1186/s12953-020-00167-3

**Published:** 2020-12-07

**Authors:** Wei Zhong Zhu, Aaron Olson, Michael Portman, Dolena Ledee

**Affiliations:** 1 Center for Integrative Brain Research, Seattle Children’s Research Institute, 1900 9th Ave, Seattle, WA 98101 USA; 2 Division of Cardiology, Department of Pediatrics, University of Washington, 1959 NE Pacific St, Seattle, Washington USA

**Keywords:** Thyroid hormone, Heart, Aged, Proteomics, Sex-based

## Abstract

**Background:**

Sex and age have substantial influence on thyroid function. Sex influences the risk and clinical expression of thyroid disorders (TDs), with age a proposed trigger for the development of TDs. Cardiac function is affected by thyroid hormone levels with gender differences. Accordingly, we investigated the proteomic changes involved in sex based cardiac responses to thyroid dysfunction in elderly mice.

**Methods:**

Aged (18–20 months) male and female C57BL/6 mice were fed diets to create euthyroid, hypothyroid, or hyperthyroid states. Serial echocardiographs were performed to assess heart function. Proteomic changes in cardiac protein profiles were assessed by 2-D DIGE and LC-MS/MS, and a subset confirmed by immunoblotting.

**Results:**

Serial echocardiographs showed ventricular function remained unchanged regardless of treatment. Heart rate and size increased (hyperthyroid) or decreased (hypothyroid) independent of sex. Pairwise comparison between the six groups identified 55 proteins (≥ 1.5-fold difference and *p* < 0.1). Compared to same-sex controls 26/55 protein changes were in the female hypothyroid heart, whereas 15/55 protein changes were identified in the male hypothyroid, and male and female hyperthyroid heart. The proteins mapped to oxidative phosphorylation, tissue remodeling and inflammatory response pathways.

**Conclusion:**

We identified both predicted and novel proteins with gender specific differential expression in response to thyroid hormone status, providing a catalogue of proteins associated with thyroid dysfunction. Pursuit of these proteins and their involvement in cardiac function will expand our understanding of mechanisms involved in sex-based cardiac response to thyroid dysfunction.

## Background

Thyroid hormone (TH) modulates myocardial function and cellular architecture through multiple mechanisms. Acting as a ligand, thyroid hormone can bind cardiomyocyte nuclear receptors and regulate transcription of genes responsible for myocardial contractility and systolic function [1, 2]. Thyroid hormone can also act extranuclear, activating cardiac ion channels and mitochondrial biogenesis [3, 4]. Disruptions in thyroid hormone homeostasis, even those defined as subclinical [5], can have substantial impact on cardiac morphology and function. Clinically, two important demographic factors independently modulate impact of thyroid dysfunction: age and sex [6]. However, as age and sex modify numerous factors, which impose cardiovascular risk, their interaction with different forms of thyroid hormone disruption are difficult to determine in a human population. The interaction between sex and age requires clarification, as thyroid hormone abnormalities occur at higher rates in elderly and females compared respectively to young and males [7, 8].

The aging population exhibits incidences for diabetes and atherosclerosis which exceed rates in younger cohorts [9]. These factors complicate interpretation of data from aging human populations with regards to thyroid hormone disturbances and its direct impact on the heart. In controlled mouse models without diabetes or atherosclerosis, aging alone promotes myocardial hypertrophy and cardiac dysfunction [10], emphasizing the importance of further examination of the impact of thyroid hormone disturbance on the aging heart.

In relation to sex and cardiovascular diseases, several studies highlight the importance of sex differences in the incidence and clinical manifestation observed between males and females [11–13]. Sex hormones and TH modulate each other through multiple mechanisms affecting gene transcription and signal transducing pathways [14–16] necessitating the need to better understand the role sex plays in modulating the impact of thyroid hormone on the aging heart. We address this issue using a proteomic approach to investigate the role sex and age play in the TH altered heart in the aged mouse.

## Methods

### Ethics statement

This investigation conforms to the *Guide for the Care and Use of Laboratory Animals* [17] and all animal protocols were reviewed and approved by Institutional Animal Care and Use Committee at Seattle Children’s Research Institute.

### Animal model

The male and female C57/BL6 mice, aged 18–20 months old, used in this study were obtained from Charles Rivers Laboratories (Wilmington, MA). The mice were divided into six groups: male euthyroid (MCON), male hypothyroid (MPTU), male hyperthyroid (MTH), female euthyroid (FCON), female hypothyroid (FPTU), female hyperthyroid (FTH). The mice were fed one of the following diets: 1) control iodine diet (TD.97350, CON), 2) hypothyroid − 0.15% propylthiouracil (PTU) diet (TD.95125), or 3) hyperthyroid - 0.1% thyroid powder (TH) diet (TD.150769) purchased from Envigo (Madison, WI). The thyroid powder (T6384) was purchased from Sigma-Aldrich (St. Louis, MO), and dosage was based on work by Thomas et al. [18]. All mice were fed for 4 weeks prior to sacrifice. Water was available *ab libitum*.

### T4 serum ELISA assays

Serum samples were collected prior to commencement of diet regime by submandibular bleeds and clearing by centrifugation. Final serum samples were collected at time of sacrifices and heart collection. The T4 levels in serum were determined using the mouse T4/Thyroxine ELISA kit (LS-F25787) from LifeSpan BioSciences, Inc., (Seattle, WA). (*n* = 5 animals/sex/treatment).

### Echocardiogram

As previously performed in our laboratory [19] serial echocardiograms were conducted prior to thyroid hormone intervention and after four weeks. Briefly, mice were initially sedated with 3% isoflurane in O_2_ at a flow of 1 LPM and placed in a supine position at which time the isoflurane is reduced to 1.5% administered via a small nose cone. ECG leads were placed for simultaneous ECG monitoring during image acquisition. Echocardiographic images were performed with a Vevo 2100 machine using a MS250 or MS550 transducer (VisualSonics, Inc., Toronto, Canada). M-Mode measurements at the midpapillary level of the left ventricle (LV) were performed at end-diastole (LVEDD) and end-systole (LVESD) to determine LV function via the fractional shortening [(LVEDD-LVESD)/LVEDD * 100] in a parasternal short axis mode for at least three heart beats. The investigators performing and interpreting the echocardiograms were blinded to the treatments. (*n* = 6–9 animals/sex/treatment).

### Sample collection

Following the final echocardiogram, mice were euthanized, the hearts extracted, rinsed in PBS buffer and frozen in liquid nitrogen. The left tibial bone was dissected out and measured using a Vernier caliper.

### 2D-gel and LC-MS analysis

Crushed heart tissue was sent to Applied Biomics (Hayward, CA) for 2D-DIGE and LC-MS/MS. In short, samples were labeled with CyDye DIGE fluors (size and charge matched) and subjected to IEF and SDS-PAGE electrophoresis. After electrophoresis, the gel was scanned using Typhoon TRIO (GE Healthcare, Chicago, IL). Each scan reveals one of the CyDye signals. The ImageQuant (version 6) (GE Healthcare, Chicago, IL) software is used to generate the image presentation data including the single and overlay images. A comparative analysis of all spots was performed using DeCyder software (version 6.5) (GE Healthcare, Chicago, IL). The protein expression ratio cut-off of 1.5-fold difference and *p* ≤ 0.1 was used in comparing same-sex control and treatment groups or males to females of same treatment. Protein spots of interest were picked from the 2D gel with the Ettan Spot Picker (Amersham Biosciences, Little Chalfont, UK). The 2D gel spots underwent in-gel trypsin digestion to generate peptides. The peptides were subjected to MALDI-TOF MS and TOF/TOF tandem MS/MS performed on an AB SCIEX TOF/TOF™ 5800 System (AB SCIEX, Framingham, MA). The resulting peptide mass and associated fragmentation spectra were submitted to GPS Explorer workstation equipped with MASCOT search engine (Matrix science) to search the database of National Center for Biotechnology Information non-redundant (NCBInr) or Swiss-Prot database. Candidates with either protein score C.I.% or Ion C.I.% greater than 95 were considered significant. An *n* = 3 animals/sex/treatment was used for these experiments. The mass spectrometry data have been deposited to the ProteomeXchange Consortium (http://proteomecentral.proteomeexchange.org) via the PRIDE [20] partner repository with the dataset identifier PXD022557.

### Protein extraction and Western blotting

Heart tissue was homogenized in RIPA buffer (20 mM Tris-HCl (pH 7.5), 150 mM NaCl, 1MM Na_2_EDTA, 0.1% SDS, 1% sodium deoxycholate, 1X protease/phosphatase inhibitor (Thermo Fisher Scientific)). Thirty micrograms of total protein extract were resolved using SDS-PAGE and electroblotted onto PVDF membranes. Standard immunoblotting protocols were followed. Horseradish peroxidase secondary antibodies were used and visualized with enhanced chemiluminescence using the ChemiDoc-It imager™ (AnalytiKjena, Germany). Membranes were stripped by washing them 2 × 15 min with 100 mM DTT, 2% (wt/vol) SDS, and 62.5 mM Tris·HCl, pH 6.7, at 70 °C, followed by three 10-min washes with TBS. Efficiency of protein transfer to PVDF membrane was determined using Pierce Reversible Protein Stain Kit for PVDF Membranes (no. 24585) (Thermo Fisher Scientific, Rockford, IL). Densitometry analyses were performed using Image J and Quantity One (Bio-Rad, Hercules, CA). All immunoblots were performed in duplicate. The primary antibodies used in this study were as follows: α 1B-glycoprotein (AF8537) purchased from R&D Systems (Minneapolis, MN) and ACAA2 (GTX115417) purchased from GeneTex (Irvine, CA). An *n* = 4 animals/sex/treatment was used for these experiments.

### Bioinformatics and motif analysis

The gene-annotation enrichment analysis software, DAVID Bioinformatics Resources 6.8 [21, 22], was utilized to acquire molecular function (MF) and KEGG (Kyoto Encyclopedia of Genes and Genomes) pathway [23] information to protein sequences obtained. A search tool for the retrieval of interacting genes/proteins (STRING, http://string-db.org/) was used to construct protein-protein interactions between the proteins. The searchable database of regulatory elements and their inferred target genes, GeneHancer embedded in GeneCards (www.genecards.org) [24, 25], was used to ascertain potential thyroid hormone receptor binding sites.

### Statistical analysis

All reported values are expressed as mean ± standard error of the mean. Significance between baseline and post-treatment values was assessed using two-way ANOVA with Tukey post hoc analysis where appropriate. One-way ANOVA was performed for values comparing treatment to same-sex control group. For immunoblots comparing control and experimental groups of a single sex we performed one-way ANOVA with Tukey post hoc analysis; and two-way ANOVA with Tukey post hoc analysis on blots containing both sexes [19]. Hypothyroid and hyperthyroid treatment groups were not compared to each other.

Criterion for significance was *p* < 0.05 for all comparisons. We preformed statistical analysis using GraphPad Prism version 7.03 (GraphPad Software).

## Results

### Serum thyroid hormone levels

**Fig. 1:**
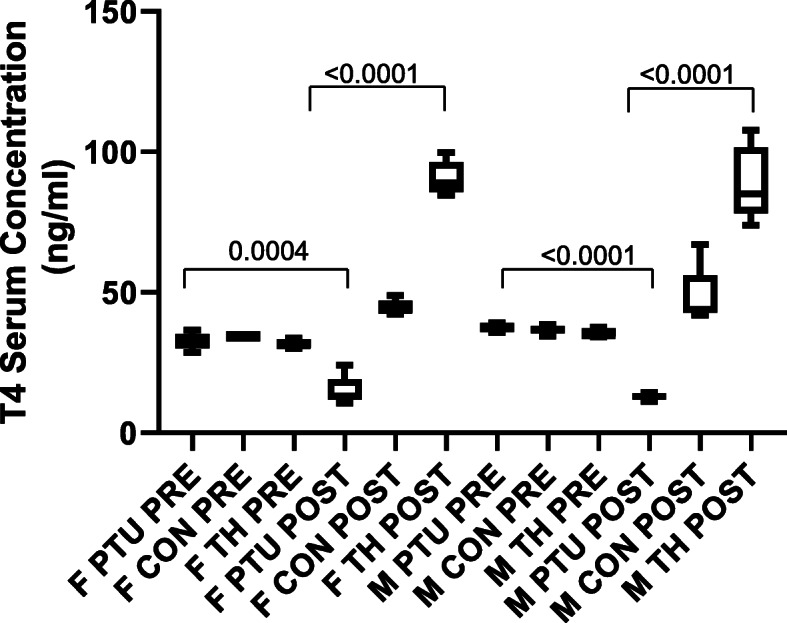
Serum T4 concentrations in aged male (M) and female (F) mice. PTU - hypothyroid mice, CON - control mice, TH - hyperthyroid mice, PRE – samples before treatment, and POST – samples after treatment. Data are presented as mean ± SEM, *n* = 6 animals/sex/treatment, two-way ANOVA followed by Tukey post hoc analysis

Efficacy of the mouse diets to induce the hypo- or hyperthyroid state was confirmed by mouse T4/thyroxine ELISA. Total T4 (TT4) serum levels were assayed prior to start and at completion of the 4-week diet regime (Fig. 1). No significant sex differences were found in the T4 serum levels of the control mice, FCON or MCON, prior to or post treatment. TH levels were altered by the diet regime. The TH deprivation diet resulted in an average decrease in TT4 levels of 17.8 ± 3.4 ng/ml and 24.5 ± 2 ng/ml in females (FPTU) and males (MPTU), respectively. Whereas, the TH diet resulted in an average increase in TT4 levels of 59 ± 2.7 ng/ml and 53.4 ± 6.1 ng/ml TT4 in females (FTH) and males (MTH), respectively.

### Morphometric and echocardiographic data

We performed serial echocardiography to assess changes in left ventricular size and function compared to baseline values (Table 1) in response to treatment. LVWPD, left ventricular posterior wall diameter, an important determinant of diastolic stiffness and pressure, significantly increased for the male by 0.42 ± 0.09 mm (*p* = 0.02) and for the female by 0.29 ± 0.08 mm (*p* = 0.042) in mice fed the hyperthyroid diet. There was no significant change observed for the other functional echocardiographic parameters between pre- and post-treatment.

**Table 1:** A. Echocardiographic and B. Morphometric analysis of the left ventricle after four weeks on prescribed diet and morphometric measurements

A	Baseline M CON	Final M CON	Baseline M PTU	Final M PTU	Baseline M TH	Final M TH	Baseline F CON	Final F CON	Baseline F PTU	Final F PTU	Baseline F TH	Final F TH
	*N* = 6		*N* = 7		*N* = 6		*N* = 8		*N* = 6		*N* = 8	
EDD, mm	3.9±0.1	4.1±0.1	4.3±0.07	4.1±0.1	4.0±0.2	4.4±0.09	4.0±0.07	3.9±0.07	3.9±0.09	4.0±0.06	3.8±0.1	4.0±0.08
ESD, mm	2.8±0.2	3.1±0.1	3.3±0.09	3.2±0.1	3.0±0.2	3.3±0.1	3.0±0.1	3.0±0.08	2.9±0.1	3.1±0.07	2.8±0.2	2.8±0.09
EF, %	50±3.6	47±3.4	48±2	46±2	53±3.7	48±2.6	49±3	45±2	49±2.4	45±2.7	54±3.7	57±2.1
FS, %	25±2.2	24±2.1	24±1	22±.9	27±2.4	25±1.5	24±2	22±1.2	25±1.5	22±1.6	28±2.4	30±1.4
LVPWd, mm	0.81±.03	0.9±0.05	0.86±0.04	0.78±0.04	0.89±0.03	1.3±0.08*	0.88±0.05	0.96±-.04	0.93±0.07	0.81±0.06	0.9±0.07	1.2±0.08*
HR, BPM	480±18	498±23	455±17	371±17**	482±21	585±21**	474±18	514±11	471±15	417±17	504±17	597±12**
B	Baseline M CON	Final M CON	Baseline M PTU	Final M PTU	Baseline M TH	Final M TH	Baseline F CON	Final F CON	Baseline F PTU	Final F PTU	Baseline F TH	Final F TH
	*N* = 8		*N* = 8		*N* = 8		*N* = 9		*N* = 9		*N* = 9	
BW, g	35±0.7	35±0.8	35±0.9	31±0.6**	34±0.9	33±0.6	29±0.6	30±1	26±0.7	25±0.3	26±0.7	29±1**
HW, mg	n/a	169±9	n/a	**137±6**^**#**^	n/a	**246±6**^**##**^	n/a	146±4	n/a	**115±3**^**##**^	n/a	**223±10**^**##**^
HW/TL (mg/mm)	n/a	9.7±0.6	n/a	**8.0±0.3**^**#**^	n/a	**14±0.4**^**##**^	n/a	8.3±0.2	n/a	**6.7±.2**^**##**^	n/a	**13±0.5**^**##**^

*CON* Euthyroid, *PTU* Hypothyroid, *TH* Hyperthyroid, *EDD* left ventricular end diastolic diameter, *ESD* left ventricular end systolic diameter, *FS* left ventricular percent fractional shortening, *EF* left ventricular percent ejection fraction, *LVPWd* left ventricular posterior wall end diastole, *HR* heart rate, *BPM* beats per minute, *BW* body weight, *HW* heart weight, *TL* tibia length. Values are means ± SEM. *N* = # of animals/sex/treatment. Bolded numbers represent significant changes. Two way ANOVA followed by Tukey post hoc analysis performed on the serial values from the same animals for EDD, ESD, EF, FS, LWPWD, HR and body weight, ^*^p < 0.05, ^**^p < 0.005; one-way ANOVA followed by Tukey post hoc analysis performed on HW and HW/TL values comparing treated group to same sex control, ^#^*p* < 0.05, ^##^*p* < 0.005

The male hypothyroid mice experienced a significant decrease of 83 ± 26 beats per minute (bpm) (*p* < 0.006) final heart rate compared to baseline; the effect did not reach significance for the females. However, both the male and female hyperthyroid mice had significantly increased heart rates. The male mice experienced a 102.8 ± 11 bpm (*p* < 0.0002), and the female mice a 92 ± 25 bpm (*p* < 0.002) increase in final heart rate compared to baseline.

Serial body weight measurements provided an assessment of systemic effect of thyroid hormone state. The male hypothyroid mice experienced a significant weight loss of 3.3 ± 0.5 g (*p* < 0.0001); whereas no change in the females was observed. In contrast, the hyperthyroid females experienced significant weight gain of 2.6 ± 0.5 g (*p* < 0.02), whereas the males remained constant.

An examination of the excised heart weight found that the male and female hypo- and hyperthyroid mice hearts had significant weight differences compared to their same sex control. The male and female hypothyroid heart weighed 33 ± 11 mg (*p* = 0.0025) and 31 ± 4 mg (*p* = 0.0021), respectively, less compared to same-sex controls. In contrast, the male and female hyperthyroid heart weighed 77 ± 8 mg (*p* = 0.0001) and 77 ± 10 mg (*p* = 0.0001), respectively, more compared to same-sex controls. The normalization of heart weight to tibia length reflected the same changes observed by heart weight measurements alone.

### Protein identification

**Fig. 2:**
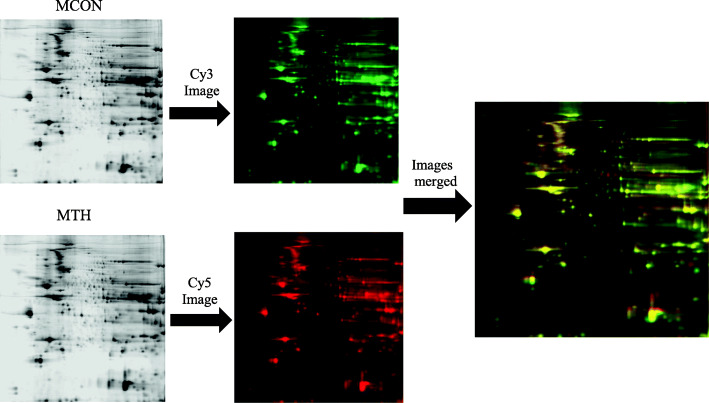
Representative images of 2D-DIGE scanned for Cy3 and Cy5 labeled proteins using Typhoon Scanner and overlaid for detection of changes in protein abundance. MCON, male control and MTH, male hyperthyroid

2D-DIGE and LC MS/MS was performed to analyze TH affected proteomic alterations in aged mouse hearts. Three biological replicates for each treatment in both sexes were used in the 2D-DIGE, allowing for the following pairwise comparisons: FCON to FPTU, FCON to FTH, MCON to MPTU, MCON to MTH, FTH to MTH and FPTU to MPTU. Image analysis of the 2D-DIGE revealed 66 protein spots showing a change in average abundance of ≥1.5 fold (*p* ≤ 0.1) between at least one of the six pairwise comparisons. Representative images of the 2D-DIGE gels is shown in Fig. 2. LC MS/MS analysis of the 66 spots identified 55 proteins (Table 2). Protein fold change ratios are depicted by a color gradient from black - representing the largest downregulation observed - to light gray – representing the largest upregulation. Hatch boxes represent no significant change in expression ratio. The largest number of proteins affected by TH status was observed between the FCON to FPTU comparison at 26/55 proteins. The FCON to FTH, MCON to MPTU, MCON to MTH comparisons each identified 15/ 55 proteins affected by TH state. The FTH to MTH comparison identified 8/55 proteins different between the sexes; and the FPTU to MPTU comparison identified 3/55. Interestingly, few of the same proteins were altered between the sexes for the same treatment.

**Table 2:**
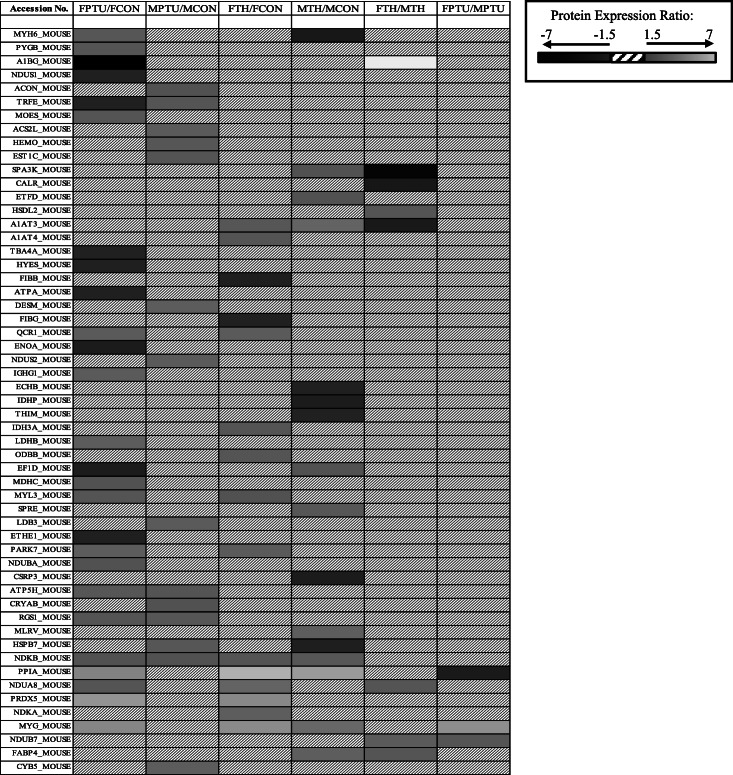
Protein Ratio of identified proteins between specified groups

*FTH* female hyperthyroid, *FCON* female control, *FPTU* female hypothyroid, *MTH* male hyperthyroid, *MCON* male control, *MPTU* male hypothyroid. Colored cells with ≥1.5-fold difference and *p*-value ≤0.1, *n* = 3 animals/sex/group

### Ontology and protein-protein interaction (PPI) network

To gain insights into molecular functions of the processes altered we performed a GO enrichment analysis using the DAVID software (Table 3). A cutoff *p* value of 0.05 was used in creating the table. The molecular functions identified for the 55 proteins by the DAVID software involved oxidoreductase and structural roles. A KEGG pathway analysis also identified predominantly cellular energetics pathways (Table 3). These results support our data as TH is known have a predominant involvement in energy metabolism and cell architecture.

**Table 3:** Enriched gene ontology (GO) terms for molecular function and KEGG pathway of identified proteins

Category	Term	Fold Enrichment	*P* Value	Bonferroni	Benjamini	FDR
Molecular Function	GO:0051287~NAD binding	35.91	6.00E-07	1.09E-04	1.09E-04	7.41E-04
	GO:0016491~oxidoreductase activity	6.54	1.30E-06	2.36E-04	1.18E-04	1.60E-03
	GO:0016616~oxidoreductase activity, acting on the CH-OH group of donors, NAD or NADP as acceptor	41.15	1.17E-04	0.02	0.01	0.14
	GO:0008137~NADH dehydrogenase (ubiquinone) activity	37.62	1.53E-04	0.03	0.01	0.19
	GO:0051539~4 iron, 4 sulfur cluster binding	32.92	2.28E-04	0.04	0.01	0.28
	GO:0003954~NADH dehydrogenase activity	75.96	6.66E-04	0.11	0.02	0.82
	GO:0051536~iron-sulfur cluster binding	20.57	9.15E-04	0.15	0.02	1.12
	GO:0008092~cytoskeletal protein binding	19.36	0.001	0.18	0.02	1.34
	GO:0000287~magnesium ion binding	8.15	0.003	0.43	0.05	3.71
	GO:0050839~cell adhesion molecule binding	13.91	0.02	0.97	0.21	21.07
	GO:0019899~enzyme binding	4.29	0.03	0.99	0.26	28.94
KEGG Pathway	mmu01100:Metabolic pathways	3.46	8.21E-09	4.93E-07	4.93E-07	8.23E-06
	mmu01130:Biosynthesis of antibiotics	10.27	9.09E-09	5.46E-07	2.73E-07	9.11E-06
	mmu05012:Parkinson's disease	11.06	9.14E-07	5.49E-05	1.83E-05	9.16E-04
	mmu00190:Oxidative phosphorylation	10.54	7.29E-06	4.37E-04	1.09E-04	7.30E-03
	mmu05010:Alzheimer's disease	8.28	3.52E-05	2.11E-03	4.23E-04	3.53E-02
	mmu05016:Huntington's disease	7.43	7.04E-05	0.004	0.001	0.07
	mmu01200:Carbon metabolism	9.51	3.42E-04	0.02	0.00	0.34
	mmu00020:Citrate cycle (TCA cycle)	22.98	6.20E-04	0.04	0.00	0.62
	mmu04932:Non-alcoholic fatty liver disease (NAFLD)	7.02	0.001	0.08	0.01	1.35
	mmu01210:2-Oxocarboxylic acid metabolism	29.02	0.004	0.23	0.03	4.37
	mmu04610:Complement and coagulation cascades	9.67	0.01	0.36	0.04	7.24
	mmu04260:Cardiac muscle contraction	9.55	0.01	0.37	0.04	7.50
	mmu01230:Biosynthesis of amino acids	9.43	0.01	0.38	0.04	7.76

**Fig. 3:**
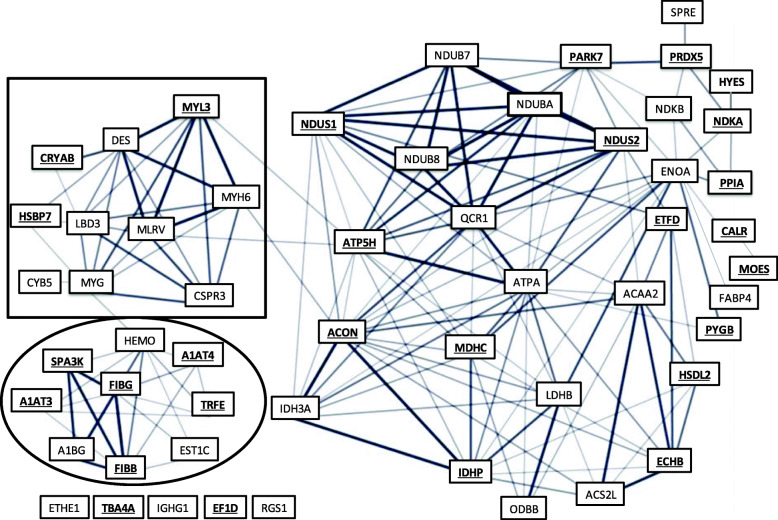
A protein-protein interaction network among the 55 identified proteins. The line thickness represents the supported confidence (correlation coefficient) between nodes. Proteins bolded and underlined possess potential THR binding sites

To explore the functional connectivity between the proteins the STRING software was used to construct a protein-protein interaction network based on an edge confidence ≥0.4 (i.e. medium to high confidence) (Fig. 3). All 55 proteins (nodes) were analyzed and 176 pairwise associations identified. Not all proteins possessed identifiable associations within this dataset with 5 proteins unlinked. The line thickness indicates the strength of data supporting the interaction between nodes. Proteins involved in energy metabolic pathways made up the largest cluster (unenclosed). Smaller clusters involved proteins associated with structural remodeling (black square) or inflammatory response roles (black circle). Of interest, the protein changes in the female hypothyroid heart represented primarily energy-metabolism proteins; whereas, the male hypothyroid proteome expressed more structural-related and inflammatory-response protein changes. In the hyperthyroid hearts, male and female, the predominant changes involved the energy-metabolism proteins.

The GeneCard database was utilized to assess if the identified proteins altered by TH status possessed potential TH receptor binding sites. The database search identified 29/55 (53%) possessing potential TH receptor binding sites (Fig. 3).

### Immunoblotting

We validated the 2D-DIGE MS/MS findings with immunoblotting for two proteins. The candidate proteins were chosen for separate reasons. A1BG (A1BG_MOUSE), alpha 1-B glycoprotein exhibited the greatest fold difference between sexes in the hyperthyroid state; and ACAA2 (THIM_MOUSE), mitochondrial 3-ketoacyl-CoA thiolase was chosen because it plays an important role in fatty acid oxidation in the heart.

**Fig. 4:**
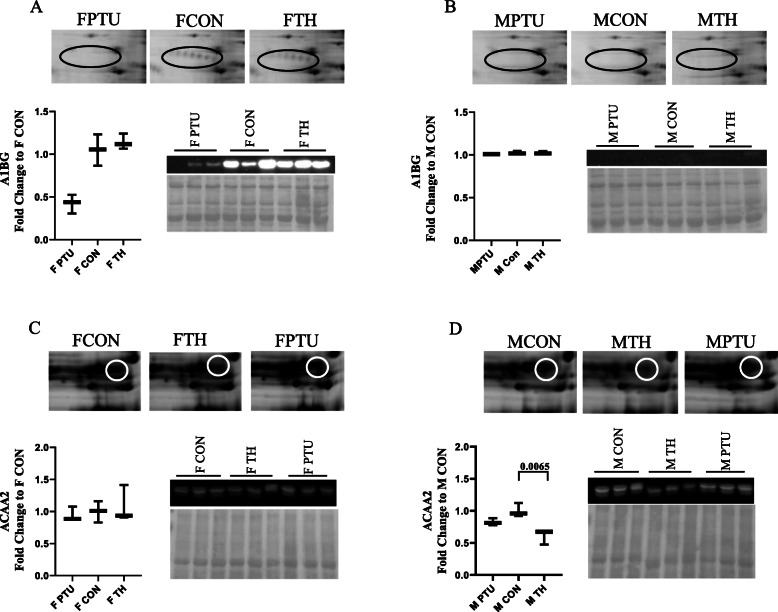
Representative 2D-DIGE and western blots of A1BG and ACAA2. **a** female A1BG, **b** male A1BG, **c** female ACAA2, and **d** male ACAA2. Top panels show the 2D-DIGE images comparing euthyroid (CON) to hypothyroid (PTU) and hyperthyroid (TH). Right-lower panel immunoblots showing protein abundance between groups. Whisker plots graph the immunoblot signal intensities presented as mean ± SEM. Statistics - one-way ANOVA followed by Tukey post hoc analysis (*n* = 4 animals/sex/treatment)

First, we examined the changes between treatments in each sex separately (Fig. 4). The top panels represent 2D-DIGE images highlighting the A1BG or ACAA2 spots. The encircled spots in Fig. 4a for FCON and FTH reveal multiple isoforms of A1BG. These spots are absent in FPTU. The A1BG spots are also absent in the male A1BG 2D-DIGE panels (Fig. 4b). The immunoblots confirmed the protein expression changes due to TH status observed by 2D-DIGE MS/MS. The FPTU heart exhibited a 2-fold A1BG expression decrease (*p* = 0.003) compared to FCON. The undetectable levels observed in the MCON mice hearts did not change with diet. Figure 4c and d show the changes in the ACAA2 protein. Like the 2D-DIGE, the ACAA2 immunoblot shows expression decreased 1.6-fold in the MTH hearts compared to MCON (*p* = 0.0065), with no observable shift in the female hearts.

**Fig. 5:**
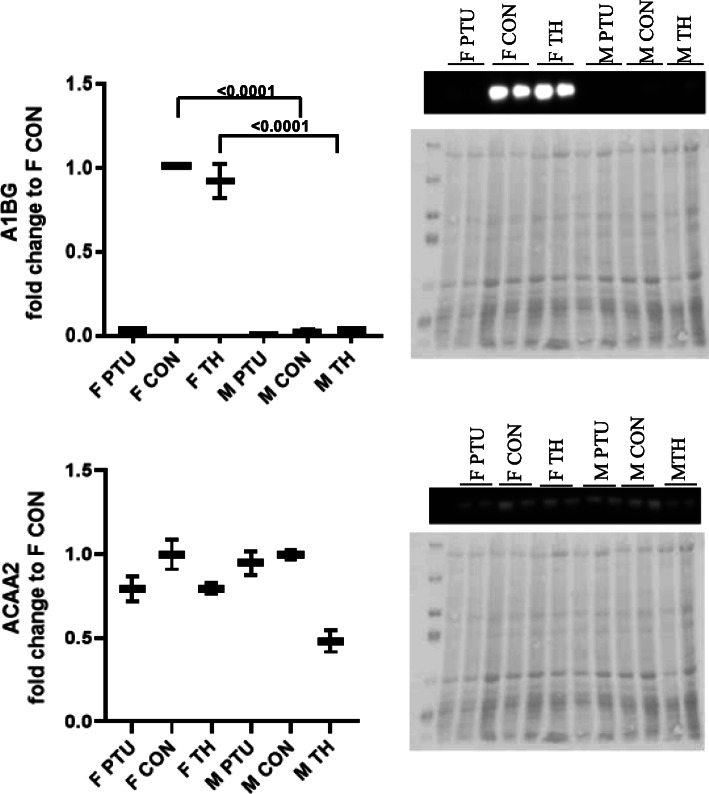
Representative western blots of expression profiles for total A1BG and ACAA2 comparing male (M) and female (F) CON, PTU and TH groups. PTU – hypothyroid, TH – hyperthyroid, and CON – euthyroid. Whisker plots of data are presented as mean ± SEM, n = 4 animals/sex/treatment, two-way ANOVA followed by Tukey post hoc analysis

For comparisons between males and females, western blots with all 6 groups (male and female: eu, hypo- and hyperthyroid) were generated (Fig. 5). There was a significant difference in expression of the A1BG protein between MCON and FCON hearts (*p* < 0.0001), due the lack of detectable A1BG in the MCON hearts. The hyperthyroid diet did not alter this disparity between male and females; however, the FPTU hearts A1BG protein levels decreased to levels observed in the MCON and MPTU hearts. ACAA2 expression was similar between MCON and FCON. The hypothyroid diet did not alter this observation, and although the MTH ACAA2 expression trended lower than the in FTH hearts it did not reach significance (*p* < 0.09).

## Discussion

We provide the first detailed proteomic analysis of thyroid impact on the heart in male and female aged mice. Previous studies evaluating thyroid effects in the hearts of elderly mice specifically excluded females or did not provide analyses according to sex [26–29]. This is an important gap in knowledge as thyroid hormone interacts with sex hormones at multiple levels ranging from transcriptional to post-translational. We show TH state elicits differential sex-based proteomic changes, especially in the female hypothyroid heart.

Thyroid hormone is known to modulate cardiac phenotype. While hyperthyroidism generally induces myocardial hypertrophy, hypothyroidism can cause a dilated cardiomyopathy [5]. These conditions are highly prevalent in aging populations [10, 30]. The impact of therapy can be highly variable, particularly on the heart [30]. Thus, the principal objective of this study was to assess the proteomic changes due to TH status in the heart for both sexes. Echocardiography allows us to make longitudinal analyses of thyroid effect on cardiac function and morphology. Both sexes appeared exquisitely sensitive to the hyperthyroid state, which induced significant elevations in heart rate. The chronotropic response occurred concomitant with increasing left posterior wall thickness, but with no change in functional parameters such as fractional shortening and ejection fraction. Although, we could not directly measure stroke volume, the increase in heart rate along with stable cardiac chamber size and ejection fraction suggests that TH induced a high cardiac output state. In contrast, the hypothyroid state caused a significant decrease in heart rate in males; however, there was limited chronotropic effect in females. With similar logic and no change in echocardiographic derived dimensions, males presumably had a low cardiac output, while females exhibited no overall change due hypothyroidism. Thus, the data suggest that the hemodynamics of the female aged hearts are more resistant to the effects of low TH levels.

Altered TT4 serum levels, including echocardiographic and morphometric data confirmed that we successfully altered thyroid state in the heart with our perturbations, establishing the basis for our proteomic analyses. We then further defined the proteome for each sex in our aging hearts according to thyroid state. Surprisingly, prior to our analysis, detailed proteomic analyses have not been performed in hearts subjected to thyroid treatment.

A prior investigation [31] reported that sex modulates TH action in both 12 and 20 months old C5/BL/7 mice. Those investigators studied effects of thyroid state on multiple systemic parameters such as body temperature and weight, as well as muscle strength and locomotor coordination. However, they did not perform end-organ specific evaluation other than heart rate. Additionally, molecular analyses were not included. Those authors also alluded to their finding of sex-based differences in TT4 serum levels in response to treatment as a potential reason for phenotypic differences. However, we found no such differences in our aged mice. The TT4 responses to treatments were virtually identical in the two sexes. This discrepancy could be related to differences in protocol; mainly as we administered daily thyronine/thyroxine in chow, and they provided T4 by intraperitoneal injection every 48 h. Regardless, sex related effects, noted in our study, would not be due to differences in serum TT4 levels.

2D-DIGE in conjunction with mass spectrometry identified 55 cardiac proteins which responded to alterations in thyroid status. Searches of pathway analyses databases, STRING and DAVID, indicated that they are involved in processes relating to mitochondrial function, structural and inflammatory responses, each of which is integral to the function and remodeling of the heart. Additionally, a search of the GeneHancer database identified 29/55 (53%) of the proteins as potential TH receptor targets. This agrees with Ayers et al. [32] report that ~ 50% of TH induced genes display THRβ binding sites.

Prior studies in our laboratory have shown that both aging and chronic hypothyroidism separately impairs myocardial fatty acid oxidation in male rats [33, 34]. Accordingly, we chose the ACAA2 protein to validate our 2D-DIGE MS/MS data given this enzyme regulates the critical final step of mitochondrial fatty acid oxidation. We found that hyperthyroidism decreased ACAA2 in aged hearts only in males. Similarly, a prior study showed decreased ACAA2 protein expression in male rat hyperthyroid liver, supporting our findings [35].

The female hypothyroid hearts appear to undergo a more dynamic response compared to their male counterparts, with 26/55 affected proteins compared 15/55 observed for the male hearts. Many of the proteins altered are members of the mitochondrial respiratory chain, such as QCR1, a subunit of the ubiquinol-cytochrome c reductase complex, and the NDUB or NDUS proteins which participate in the mitochondrial membrane respiratory chain NADH dehydrogenase (Complex 1). This together with our physiological data suggest the female aged heart is more accommodating to the hypothyroid state. Therefore, the finding of more metabolic proteins undergoing altered expression in female hypothyroid heart may yield insight into potential mechanistic difference between sexes.

Inflammatory response proteins were also altered by thyroid status, many of which are glycoproteins. For example, A1BG, a novel thyroid hormone target, is a plasma glycoprotein of unknown function. Glycoproteins play key roles in inflammatory diseases, are known to be altered in cardiomyopathies and thyroid disorders, and have the potential to be exploited for diagnosis and/or treatment targets [36, 37]. Glycoproteins identified in this study, such as Serpina3K (SPA3K) and A1BG, have been shown to be differentially expressed in myocardial pathology [38, 39]. In this study A1BG protein was absent in the MCON hearts and highly expressed in the FCON. The FPTU A1BG expression plunged to the non-detectable levels of MCON and MPTU. Thyroid dysfunction is primarily diagnosed by the levels of thyroid stimulating hormone (TSH), a regulator of TH production. However, bioactivity of TSH is related to glycosylation modifications and age [40]; current clinical assays do not distinguish between TSH glycosylated forms. The expression of varied modified TSH protein in the aged population may account for discrepancies in diagnosis and prevalence observed in studies of aging patients, emphasizing the need for identifying other diagnostic targets. Xiao et al. reported on zinc-α2- glycoprotein increase and its correlation with TH levels in hyperthyroid patients; however, that study did not analyse male and females separately [41]. We have identified several glycoproteins altered in the aged mouse by TH state and differentially expressed between the sexes. Hence, our findings show the importance of examining both males and females for the identification and use of potential biomarkers towards better discernment of subclinical thyroid states or indications for treatment.

TH regulates cardiovascular remodelling, affecting size, shape and function in the heart [42, 43]. Our study identified several cardiac remodelling proteins further validating our data. Thyroid disorders affect the myocardium similarly to that observed by the aging process, such as changes to cardiac contractility, cardiac output and systemic vascular resistance [44]. Additionally, it is well recognized that structural and functional differences exist between male and female, such as anatomical size [45] and electrical activity [46]. We identified myosin heavy chain α (MHC6), for instance, a major protein in cardiac muscle and contractile function that is known to be regulated by TH via the TH hormone nuclear receptor sites in its promoter region [47]. Other structural proteins identified included regulatory myosin light chain 2 and 3 which interact directly with MHC6. Structure-related proteins identified, such as alpha B-crystallin interact with desmin/ actin cytoskeletal complexes, and are predicted to play a protective role during stress conditions [48]. The structural proteins identified in addition to being altered by TH state also showed preferential sex expression. Further investigations of these proteins and their cellular interactions will aid in better understanding any sexual dimorphisms.

An interesting disparity was observed in our study in that the echocardiographic measurements showed modest phenotypic differences between males and females compared to the key differences observed for the proteomic changes. One explanation is that the proteins we observed altered by TH status are predominantly involved in myocardial substrate utilization as opposed to structural or hypertrophic changes. Supporting this rationale, previous work in our lab showed TH treated aged male mouse heart experienced significant alterations in substrate utilization with fatty acids, but exhibited limited or unchanged cardiac functions compared to baseline or aged untreated [31, 33, 34]. Thus, we would need to do specific studies on cardiac substrate utilization for fatty acids to define these sex-differences.

### Limitations of the study

First, the proteins involved in thyroid hormone regulation identified by the 2D-DIGE and LC-MS/MS method used in this study are by no means meant to be an exhaustive list. Second, as a screening study, it is not within the scope of the present research to determine if the changes in protein expression levels are due to direct or indirect modulation of the proteins by thyroid hormone. Third, small sample size limits the statistical power of our data [49]. Fourth, it is important to note a major difference between aging female mice and postmenopausal aged women; aging female mice do not have the equivalent of menopause. Aging female mice become acyclic by 11–16 months and develop estrogen deficiency but not as profoundly as aged women [50].

## Conclusions

In this study we demonstrate that thyroid hormone differentially regulates the cardiac proteomic profile in an age and sex influenced manner. Although thyroid dysfunction is more prevalent in women previous animal studies have excluded females. In our study, the aged female heart had a greater number of proteomic changes in response to thyroid hormone state. The identification of numerous sex-based differentially expressed proteins altered by thyroid hormone status shows the importance of examining both sexes. The integration of sex-specific data will yield a better understanding of thyroid hormone regulation in the aged heart.

## Data Availability

All data used to support the findings of this study are available upon request to the corresponding author. The dataset generated in this study has been deposited in the ProteomeXchange Cosortium (http://proteomecentral.proteomeexchange.org) via the PRIDE partner repository with the dataset identifier PXD022557.

## Abbreviations

TH: Thyroid hormone
MCON: Male euthyroid
MPTU: Male hypothyroid
MTH: Male hyperthyroid
FCON: Female euthyroid
FPTU: Female hypothyroid
FTH: Female hyperthyroid
ECG: Echocardiogram
2D-DIGE: Two dimensional difference gel electrophoresis
LC-MS/MS: liquid chromatography with tandem mass spectrometry
A1BG: Alpha 1-B glycoprotein
ACAA2: Mitochondrial 3-ketoacyl-CoA thiolase
